# *Eucommia ulmoides* Leaf Polyphenols Alleviate Hypertension and Modulate Gut Microbiota and Metabolic Profiles in Spontaneously Hypertensive Rats

**DOI:** 10.3390/foods15173032

**Published:** 2026-08-27

**Authors:** Jia Song, Delong Ma, Yaqi Fan, Rongyu Zhao, Chenyu Yang, Zhenhua Su, Yu Zheng, Min Wang

**Affiliations:** State Key Laboratory of Bio-Based Fiber Materials, School of Biological Engineering, Tianjin University of Science & Technology, Tianjin 300457, China; tjsongjia@tust.edu.cn (J.S.); madelong@mail.tust.edu.cn (D.M.); 15234131468@163.com (Y.F.); 15222757512@163.com (R.Z.); 13271220117@163.com (C.Y.); suzhenhua@tust.edu.cn (Z.S.); yuzheng@tust.edu.cn (Y.Z.)

**Keywords:** *Eucommia ulmoides* leaf, polyphenols, hypertension, gut microbiota, metabolomics, short-chain fatty acids

## Abstract

*Eucommia ulmoides* leaves, a traditional edible and medicinal resource, are a rich source of bioactive polyphenols. In this study, male Wistar rats served as the normal control, while spontaneously hypertensive rats were randomized into model, positive control (nifedipine, 6.0 mg/kg/d), and low-, medium-, and high-dose groups receiving 1.25, 2.50, and 3.75 mL/kg/d of the polyphenol extract (*n* = 8/group) via intragastric gavage for 4 weeks. The extract supplementation significantly reduced systolic and diastolic blood pressure to approximately 166.33 mmHg and 113.93 mmHg, respectively, thereby improving vascular function (*p* < 0.05), accompanied by alleviation of lipid metabolism disorders and oxidative stress. In addition, it mitigated intestinal injury and enhanced gut barrier integrity. Notably, it reshaped the gut microbiota by enriching beneficial bacteria and suppressing harmful taxa, which is associated with increased production of short-chain fatty acids. Integrated metabolomics analysis further demonstrated that it modulated multiple metabolic pathways, particularly arachidonic acid and tryptophan metabolism, which are closely linked to vascular homeostasis, inflammation, and oxidative stress. Collectively, these findings indicate that the antihypertensive effects of the polyphenol extract are potentially linked to the regulation of the gut microbiota–metabolite axis, highlighting its promising application as a functional food ingredient for cardiovascular health.

## 1. Introduction

Hypertension is a leading risk factor for cardiovascular diseases (CVDs) worldwide [1,2]. With the acceleration of global population aging, the prevalence of hypertension continues to increase, posing a major public health challenge [3,4]. In recent years, the concept of the “gut–heart axis” has provided a novel perspective for the prevention and management of hypertension [5]. Accumulating evidence suggests that hypertension is closely linked to gut microbiota dysbiosis [6,7]. For instance, a significant reduction in gut microbial diversity, along with an enrichment of *Prevotella*, has been observed in hypertensive patients [8]. In spontaneously hypertensive rats (SHRs), gut microbiota imbalance is characterized by an increased Firmicutes/Bacteroidetes (F/B) ratio, impaired intestinal barrier function, and elevated blood pressure. Furthermore, gut microbial dysbiosis has been shown to impair intestinal barrier integrity, as evidenced by the decreased expression of tight junction proteins (e.g., ZO-1 and Occludin), and to promote chronic inflammation, thereby contributing to vascular endothelial dysfunction [9]. Therefore, dietary interventions targeting the gut microbiota have emerged as a promising strategy for the prevention of hypertension and its associated target organ damage [10,11].

Among various dietary bioactive compounds, polyphenols and flavonoids have attracted considerable attention due to their potent antioxidant and vasoprotective effects [12,13]. Increasing evidence indicates that quercetin supplementation exerts significant antihypertensive effects. In spontaneously hypertensive rats (SHRs), quercetin at a dose of 100 mg/kg has been shown to significantly decrease systolic blood pressure, while daily supplementation of 500 mg for 12 weeks reduced systolic blood pressure by approximately 6.55 mmHg in patients with type 2 diabetes and hypertension [14]. Similarly, total flavonoids derived from *Astragalus complanatus* at a high dose of 200 mg/kg have been demonstrated to significantly lower mean arterial pressure in SHR [15]. Flavonoids from sea buckthorn seeds have also been reported to improve lipid metabolism under hypertensive conditions [16].

Despite these well-documented antihypertensive effects of natural products, their underlying mechanisms remain incompletely understood. Plant-derived polyphenols exhibit remarkable structural diversity, contributing to their complex biological activities, while current mechanistic studies remain relatively limited. Existing evidence has primarily focused on classical pathways; for instance, the antihypertensive effects of polyphenols have been largely attributed to angiotensin-converting enzyme (ACE) inhibition [17]. In contrast, some studies have mainly emphasized clinical blood pressure changes and macroscopic phenotypes [18], with insufficient integration of the gut microbiota–metabolite–host regulatory axis.

*Eucommia ulmoides* leaves, a traditional edible–medicinal resource in China, are rich in chlorogenic acids and flavonoids and have been reported to exhibit antihypertensive activity [19,20]. In spontaneously hypertensive rats (SHRs), *E. ulmoides* leaves have been shown to improve vascular relaxation by increasing nitric oxide (NO) levels [21]; their blood pressure-lowering and hemodynamic benefits have also been demonstrated [22]. More recently, evidence suggests that the antihypertensive effects of *E. ulmoides* may involve modulation of the gut microbiota [23]. However, the overall mechanisms of *Eucommia ulmoides* leaf polyphenols (EULP) remain incompletely elucidated, particularly regarding gut microbiota remodeling, key metabolites such as short-chain fatty acids (SCFAs), and multi-omics integration. Most previous studies have attributed their effects to direct action on vascular smooth muscle, with limited investigation of in situ intestinal biotransformation and the consequent ecological shifts [24]. In particular, the associations among EULP, gut microbiota alterations, SCFA production, and blood pressure regulation remain incompletely understood.

Based on this rationale, the present study aimed to investigate the antihypertensive effects of EULP in spontaneously hypertensive rats (SHRs) and to elucidate the underlying mechanisms involving the gut microbiota–metabolite axis by employing an experimental model integrating 16S rRNA sequencing, global metabolomics analysis, and targeted quantification of short-chain fatty acids (SCFAs) to systematically evaluate the antihypertensive effects of EULP and elucidate its regulatory mechanisms on gut microbiota and SCFA metabolism. This study not only provides a comprehensive understanding of the multi-target mechanisms underlying EULP-mediated blood pressure reduction but also establishes a foundation for developing functional food products that target gut microbiota.

## 2. Materials and Methods

### 2.1. Materials and Reagents

Air-dried Eucommiae Folium was purchased from Hebei Jiaheng Chinese Herbal Medicine Co., Ltd. (Anguo, Hebei, China).

Gallic acid standard and Folin–Ciocalteu reagent were obtained from Beijing Solarbio Science & Technology Co., Ltd. (Beijing, China). Acetonitrile and methanol were supplied by Tianjin Jiangtian Chemical Co., Ltd. (Tianjin, China). Nitric oxide (NO) assay kits were purchased from Nanjing Jiancheng Bioengineering Institute (Nanjing, China). ELISA kits for endothelial nitric oxide synthase (eNOS), angiotensin II (Ang II), endothelin (ET), norepinephrine (NE), prostaglandin E2 (PGE2), prostacyclin (PGI2), endothelium-derived hyperpolarizing factor (EDHF), and aldosterone (ALD), as well as assay kits for total cholesterol (TC), triglycerides (TG), low-density lipoprotein cholesterol (LDL-C), high-density lipoprotein cholesterol (HDL-C), superoxide dismutase (SOD), glutathione peroxidase (GSH-Px), and malondialdehyde (MDA), were obtained from Shanghai Enzyme-linked Biotechnology Co., Ltd. (Shanghai, China). A bicinchoninic acid (BCA) protein assay kit was purchased from Beyotime Biotechnology (Shanghai, China). Oasis PRiME HLB solid-phase extraction cartridges (6 cc/200 mg) were purchased from Waters Corporation (Milford, MA, USA).

### 2.2. Instruments and Equipment

An electronic analytical balance (CP1502) was obtained from Mettler-Toledo (Shanghai, China). A high-speed centrifuge (TG16-WS) was supplied by Xiangyi Laboratory Instrument Development Co., Ltd. (Changsha, Hunan, China). A multifunctional microplate reader (SYNERGY GY4) was purchased from BioTek Instruments, Inc. (Winooski, VT, USA). A rotary evaporator was obtained from Heidolph (Schwabach, Germany). A circulating water vacuum pump (SHZ-D) was supplied by Zhengzhou Kairui Instrument Equipment Co., Ltd. (Zhengzhou, China). A non-invasive blood pressure measurement system for rodents (Medlab) was purchased from Nanjing Calvin Biotechnology Co., Ltd. (Nanjing, China). A UV–Vis spectrophotometer (UVmini-1240) was obtained from Shimadzu Corporation (Kyoto, Japan) and a gas chromatography–mass spectrometry system (GC-MS, TQ8050NX) were obtained from Shimadzu Corporation (Kyoto, Japan).

### 2.3. Animal Experiment

Ten-week-old specific pathogen-free (SPF) male Wistar rats (180–220 g) and male spontaneously hypertensive rats (SHR, 10 weeks old, 200 ± 20 g, SPF grade) were purchased from SPF Biotechnology Co., Ltd. (Beijing, China). All animal experiments were conducted in accordance with the protocol approved by the Institutional Animal Health Committee of Tianjin University of Science and Technology (SCXK2025-0007).

Wistar rats were assigned to the normal control group (NC). SHRs were randomly divided into five groups: model group (Model), positive control group (PC), low-dose EULP group (LD), medium-dose EULP group (MD), and high-dose EULP group (HD), with eight rats in each group (*n* = 8). All animals were housed in the Animal Experimental Center of the School of Biotechnology, Tianjin University of Science and Technology, under controlled environmental conditions. After a 5-day acclimation period, rats received daily intragastric administration for 4 weeks as follows: NC group, distilled water; Model group, distilled water; PC group, nifedipine (6.0 mg/kg/day) [25,26]; LD group, EULP (1.25 mL/kg/day); MD group, EULP (2.50 mL/kg/day); and HD group, EULP (3.75 mL/kg/day). The EULP preparations were adjusted to the corresponding concentrations to ensure an identical final gavage volume among the treatment groups. The NC, Model, and PC groups also received the same final administration volume.

### 2.4. Preparation of Eucommia ulmoides Leaf Aqueous Extract

The optimal extraction conditions for *Eucommia ulmoides* leaves were previously established in our laboratory as follows: a solid–liquid ratio of 1:25 (g/mL), extraction temperature of 84 °C, extraction time of 66 min, and two extraction cycles. Under these conditions, the aqueous extract of *E. ulmoides* leaves was prepared and collected for subsequent experiments. The prepared aqueous extract was stored at −20 °C until use within one month. Before each administration, the extract was thawed and thoroughly mixed.

### 2.5. Determination of Total Polyphenol Content

The total polyphenol content was determined using the Folin–Ciocalteu method [27]. Gallic acid was used as the standard to establish the calibration curve (y = 6.46x + 0.014, R^2^ = 0.9982) within a linear range of 0–0.10 mg/mL. After appropriate dilution, the total polyphenol content of the extract was determined to be 20.70 ± 0.02 mg/mL.

### 2.6. Measurement of Blood Pressure, Heart Rate, and Body Weight

During the experimental period, the body weight of rats in each group was recorded at regular intervals. Systolic blood pressure (SBP), diastolic blood pressure (DBP), and heart rate (HR) were measured using a non-invasive tail-cuff system under conscious and calm conditions. Before measurement, rats were acclimated to room temperature (25 ± 1 °C), and all procedures were performed gently to minimize stress. After the animals remained stable, a tail cuff was placed at the base of the tail, and pressure was increased at a rate of 20 mmHg/s. After pulse disappearance, the pressure was further increased by 20 mmHg, followed by gradual deflation. Measurements were repeated at 2 min intervals. Three consecutive valid readings with heart rate variation ≤ 10 beats/min and blood pressure variation ≤ 6 mmHg were selected, and the average value was calculated as the final result.

### 2.7. Determination of Serum Biochemical, Lipid Metabolism, and Oxidative Stress Indicators

After fasting for 12 h, rats in each group were weighed and anesthetized by intraperitoneal injection of 10% chloral hydrate. Blood samples were collected by cardiac puncture and centrifuged at 3000× *g* for 15 min. The upper serum layer was carefully collected and stored at −80 °C until further analysis. Serum levels of NO, eNOS, Ang II, ET, NE, PGE2, PGI2, EDHF, and ALD, as well as TC, TG, LDL-C, HDL-C, SOD, GSH-Px, and MDA, were determined using the corresponding commercial assay kits according to the manufacturers’ instructions. Absorbance was measured using a multifunctional microplate reader (SYNERGY GY4, BioTek Instruments, Inc., Winooski, VT, USA), and the concentrations or activities were calculated according to the manufacturers’ instructions.

### 2.8. Determination of Cardiac Inflammatory Protein Expression

Heart tissues (0.1 g) from the NC, Model, PC, LD, and HD groups were homogenized with 4–6 grinding beads in 900 μL precooled RIPA lysis buffer containing 9 μL PMSF. The homogenates were centrifuged at 4500 rpm for 15 min at 4 °C, and the supernatants were collected and stored at −80 °C. Protein concentrations were determined using a BCA protein assay kit. Protein samples were mixed with sample loading buffer at a ratio of 4:1 (sample:buffer), boiled at 100 °C for 10 min, cooled to room temperature, and stored at −20 °C until use.

Proteins were separated by SDS-PAGE at 80 V for 30 min and subsequently at 120 V until the bromophenol blue dye front reached the bottom of the gel, followed by transfer to PVDF membranes. Membranes were blocked with 5% non-fat milk for 1.5 h at room temperature and incubated overnight at 4 °C with primary antibodies against p-NF-κB p65, NF-κB p65, p-IκBα, IκBα, IL-1β, TNF-α, and β-actin. After washing three times with TBST (5 min each), membranes were incubated with fluorescent secondary antibodies for 2 h at room temperature and washed three times with TBST. Protein bands were visualized using an Odyssey infrared imaging system and quantified using ImageJ 1.53.

### 2.9. Histopathological Analysis

Rats were sacrificed by cervical dislocation under anesthesia and dissected. Ileum and heart tissues were collected and fixed in 4% paraformaldehyde. After routine paraffin embedding and sectioning, tissue sections were deparaffinized, rehydrated, and stained with hematoxylin and eosin (H&E). Following dehydration, clearing, and mounting, histopathological changes were observed and imaged under a light microscope at 200× magnification.

### 2.10. 16S rRNA Gene Sequencing and Short-Chain Fatty Acid Analysis

Fecal samples from the NC, Model, LD, and HD groups were collected at the end of the experiment and immediately stored at −80 °C. Microbial genomic DNA was extracted, and DNA quality, concentration, and purity were assessed by 1% agarose gel electrophoresis and NanoDrop 2000 (Thermo Scientific, Waltham, MA, USA). The bacterial 16S rRNA gene was amplified by PCR using barcode-containing primers. PCR products were recovered from 2% agarose gels, purified using a PCR Clean-Up Kit (Yuhua, China), and quantified using a Qubit 4.0 fluorometer (Thermo Scientific, Waltham, MA, USA). Sequencing libraries were constructed and sequenced on an Illumina HiSeq 2000 platform (Illumina, Inc., San Diego, CA, USA). Raw sequencing data were quality-filtered to obtain high-quality sequences. Alpha diversity, beta diversity, and taxonomic composition were analyzed using the Majorbio Cloud Platform (Shanghai Majorbio Bio-Pharm Technology Co., Ltd., Shanghai, China). Microbial functional profiles were predicted using PICRUSt2 (v2.2.0) based on the 16S rRNA sequencing data, with functional annotation against the KEGG and COG databases. SCFA concentrations were determined using the same platform.

### 2.11. Serum Metabolomics Analysis

Serum samples collected at the end of the animal experiment were stored at −80 °C until metabolomic analysis. Serum metabolomic profiling was performed by Majorbio Bio-Pharm Technology Co., Ltd. (Shanghai, China) using an LC-MS-based untargeted metabolomics platform. Sample preparation, LC-MS data acquisition, data preprocessing, and metabolite annotation were performed according to the standardized analytical workflow of the platform. Multivariate analyses, including principal component analysis (PCA) and partial least squares discriminant analysis (PLS-DA), were performed to evaluate overall metabolic differences among groups. Differential metabolites were identified using VIP > 1.0 and *p* < 0.05. Key differential metabolites were further screened using VIP > 2.0. Hierarchical clustering, volcano plot analysis, metabolite classification, and KEGG pathway enrichment analysis were subsequently performed.

### 2.12. Identification of Polyphenolic Compounds in Eucommia ulmoides Leaf Extract

Sample preparation and solid-phase extraction: Appropriate amounts of *E. ulmoides* leaf aqueous extract were loaded onto Oasis PRiME HLB cartridges (6 cc/200 mg). The cartridges were washed with 4 mL of 5% methanol in water and eluted with 4 mL of acetonitrile/methanol (90:10, *v*/*v*).

Gas chromatography-mass spectrometry (GC-MS) conditions: GC analysis was performed using an HP-5 capillary column (30 m × 250 μm × 0.25 μm). The injector temperature was set at 300 °C, and helium was used as the carrier gas. The oven temperature program was as follows: initial temperature of 100 °C, increased to 280 °C at 3 °C/min, with a total run time of 60 min. MS detection was conducted in the mass range of 50–700 *m*/*z* at a scan rate of 20,000 amu/s, with a data acquisition rate of 33.3 s^−1^. The ion source temperature was set at 200 °C, and ionization was performed using electron impact (EI) at 70 eV. The acquired EI mass spectra were compared with reference spectra in the NIST mass spectral library for compound annotation, and the detected compounds were tentatively identified based on the spectral-library matching results.

### 2.13. Statistical Analysis

All data were expressed as mean ± standard deviation (mean ± SD). The normality of data distribution was assessed using the Shapiro–Wilk test. Statistical analyses were performed using IBM SPSS Statistics 27.0. Differences among multiple groups were evaluated using one-way analysis of variance (ANOVA), followed by Tukey’s multiple comparisons test when the ANOVA indicated a significant difference. A two-sided *p* < 0.05 was considered statistically significant. Spearman correlation analysis was performed to assess the relationships among gut microbiota, differential metabolites, and hypertension-related indicators. Data plotting and visualization were performed using Origin 2018 and Adobe Illustrator 2025. Correlation results were visualized using Cytoscape 3.10.0.

## 3. Results

### 3.1. Effects of EULP on Blood Pressure, Heart Rate, and Body Weight in SHRs

**Figure 1:**
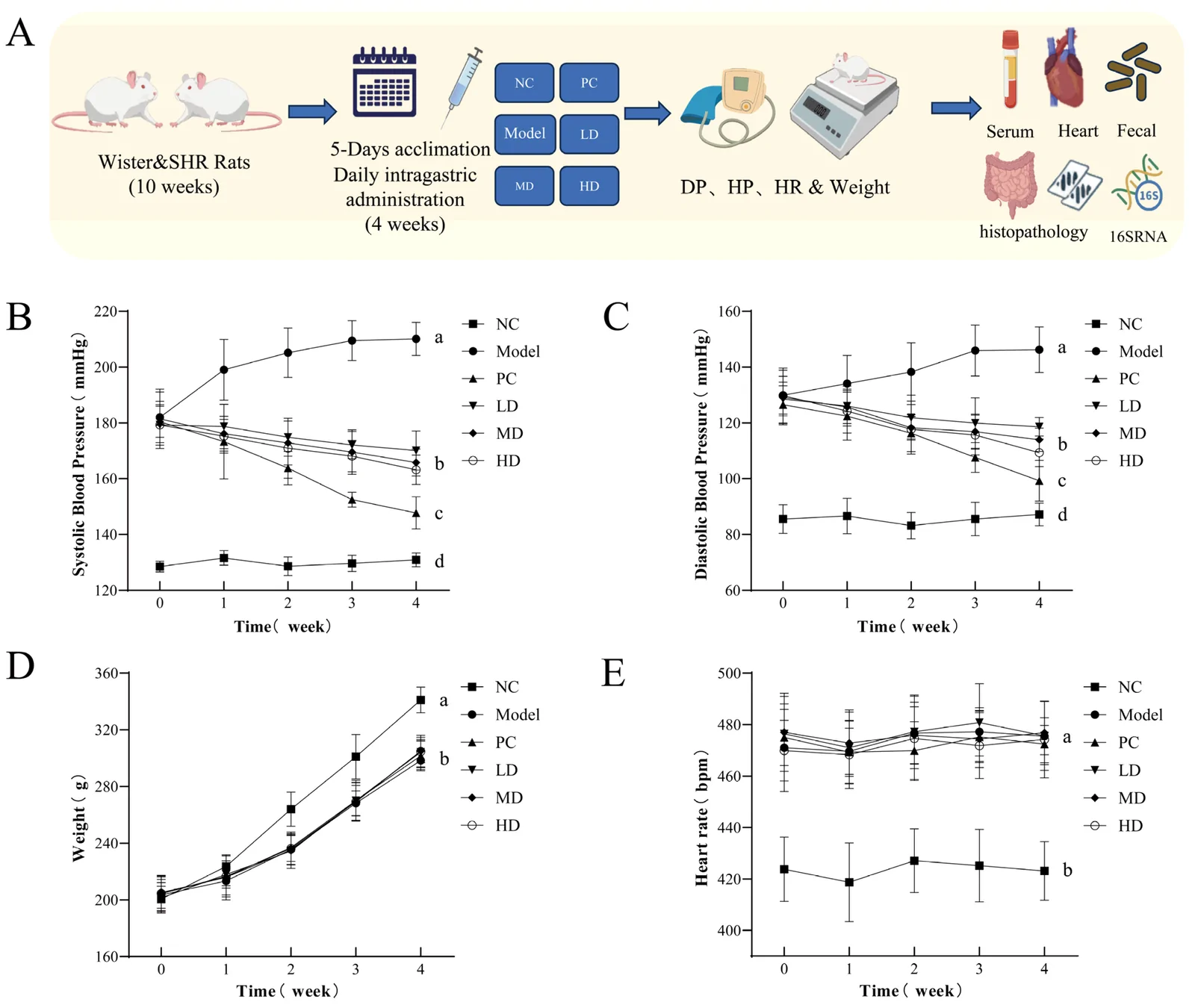
Effects of EULP on blood pressure and physiological parameters in SHRs. (**A**) Experimental workflow of EULP intervention and sample collection. (**B**,**C**) Systolic (SBP) and diastolic blood pressure (DBP) during the intervention period. (**D**) Heart rate. (**E**) Body weight. Data are expressed as mean ± SD (*n* = 8). Different lowercase letters (a, b, c, d) indicate significant differences among groups at week 4 (*p* < 0.05). Groups sharing the same letter are not significantly different.

The changes in systolic blood pressure (SBP), diastolic blood pressure (DBP), heart rate (HR), and body weight in each group were dynamically monitored (Figure 1). During the experimental period, blood pressure in the NC group remained stable. In contrast, SBP and DBP in the Model group increased progressively with age and reached (210.12 ± 5.93) mmHg and (146.22 ± 8.18) mmHg, respectively, at week 4, which were significantly higher than those in the NC group (*p* < 0.05), indicating the successful establishment of the hypertensive model. After 4 weeks of intervention, SBP and DBP in the EULP-treated groups were maintained at approximately 166.33 mmHg and 113.93 mmHg, respectively. Notably, the MD and HD groups exhibited more pronounced antihypertensive effects (*p* < 0.05), indicating a significant antihypertensive effect of EULP in SHRs.

Heart rate monitoring showed that the HR in the Model group was significantly higher than that in the NC group both before and during the experiment (*p* < 0.05), which is consistent with the characteristic sympathetic hyperactivity observed in SHRts [28]. Compared with the Model group, no significant differences in HR were observed in the EULP-treated groups (LD, MD, and HD) (*p* > 0.05), suggesting that the antihypertensive effects of EULP were independent of heart rate regulation.

In addition, body weight measurements indicated that, except for the NC group, rats in other groups exhibited relatively lower body weight at the initial stage of the experiment. However, no significant differences in body weight gain were observed among the Model, PC, and EULP-treated groups (LD, MD, and HD) throughout the experimental period (*p* > 0.05), excluding the potential confounding effect of body weight on the experimental outcomes.

### 3.2. Effects of EULP on Cardiac Histopathology in Rats

**Figure 2:**
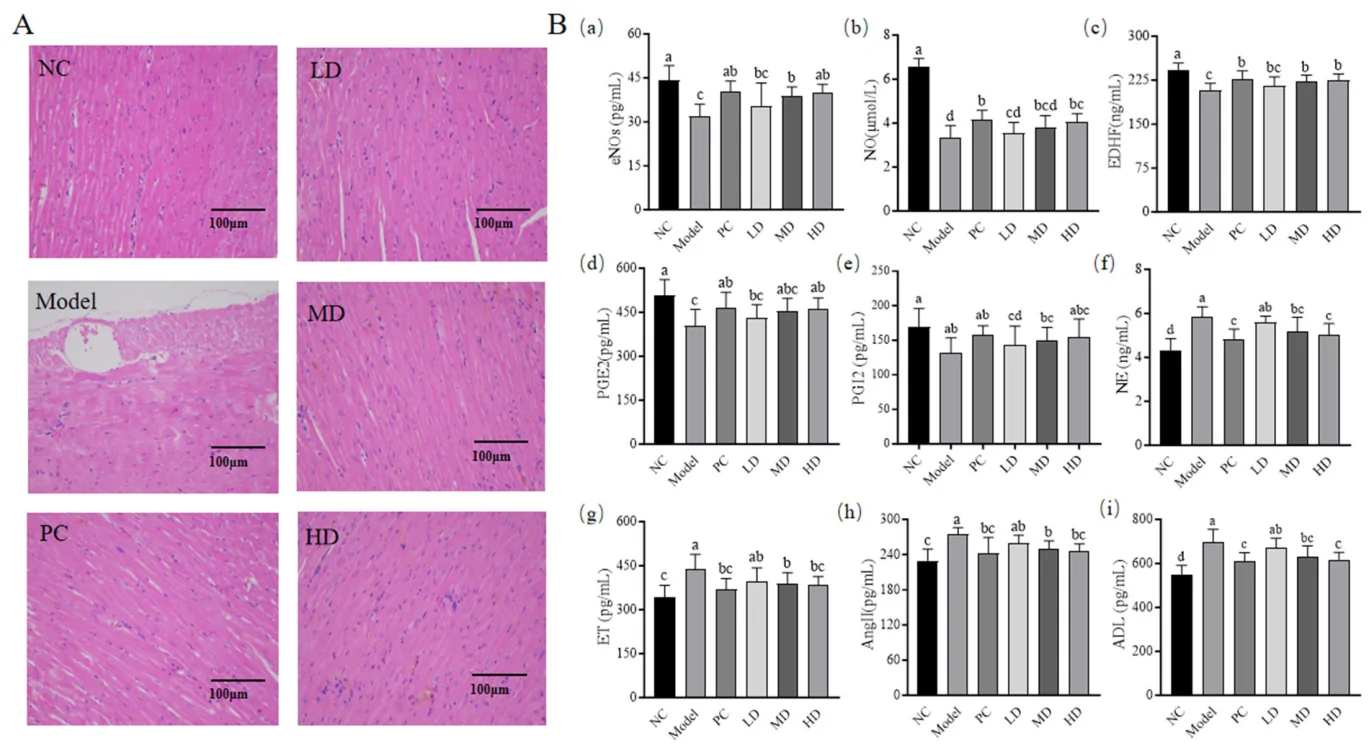
Effects of EULP on cardiac histopathology and serum vasoactive factors in SHRs. (**A**) Representative H&E-stained myocardial sections (scale bar = 100 μm). (**B**) Serum levels of vasoactive factors, including (a) eNOS, (b) NO, (c) EDHF, (d) PGE2, (e) PGI2, (f) NE, (g) ET-1, (h) Ang II, and (i) ALD. Data are presented as mean ± SD (*n* = 6). Different lowercase letters indicate significant differences among groups (*p* < 0.05).

Histopathological analysis based on hematoxylin and eosin (H&E) staining (Figure 2A) revealed that myocardial fibers in the NC group were orderly arranged with clear structural integrity and no obvious pathological alterations. In contrast, the Model group exhibited significant myocardial damage, characterized by disorganized myocardial fibers, partial cardiomyocyte swelling, and enlarged intercellular spaces.

Compared with the Model group, the PC group showed noticeable improvement in cardiac tissue morphology, with more regular myocardial fiber arrangement and alleviated cellular swelling. Similarly, all EULP-treated groups (LD, MD, and HD) displayed varying degrees of amelioration in myocardial injury. Among them, the MD and HD groups exhibited more pronounced protective effects, as evidenced by relatively well-organized myocardial fibers and cellular structures approaching those of the NC group. These findings indicate that EULP not only lowers blood pressure but also protects against hypertension-induced cardiac injury, indicating its potential cardioprotective properties.

### 3.3. Effects of EULP on Serum Vascular Factors in Rats

To evaluate the effects of EULP on vasoactive factors associated with vascular relaxation and contraction, the serum levels of vasodilatory factors eNOS, NO, EDHF, PGE2, and PGI2, as well as vasoconstrictive factors NE, ET, Ang II, and ALD, were measured in SHRs. As shown in Figure 2B, compared with the NC group, serum levels of NO and eNOS were significantly decreased in the Model group SHRs (*p* < 0.05). After 4 weeks of EULP intervention, all dose groups showed varying degrees of increase in NO and eNOS levels, with the most significant increases observed in the MD and HD groups (*p* < 0.05). Concurrently, compared with the NC group, serum levels of ET, NE, Ang II, and ALD were significantly elevated in the Model group of SHRs (*p* < 0.05). Following EULP intervention, all dose groups exhibited a decreasing trend in the levels of these vasoconstrictor factors, demonstrating a certain dose-dependency. The most prominent reductions were observed in the MD and HD groups (*p* < 0.05).

### 3.4. Effects of EULP on Serum Lipid Metabolism and Oxidative Stress Indicators in SHR

**Figure 3:**
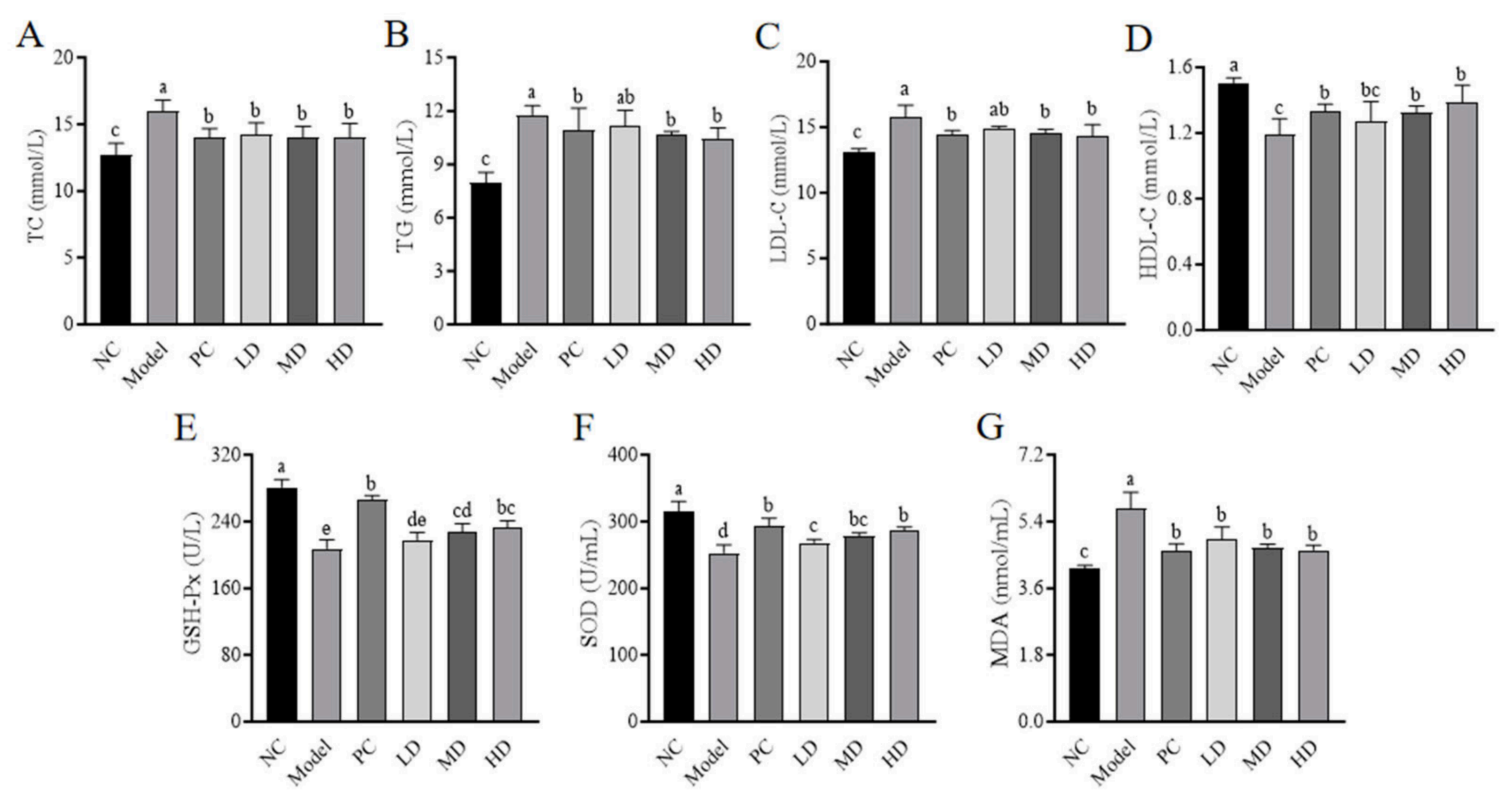
Effects of EULP on lipid profiles and oxidative stress markers in SHRs. (**A**–**D**) Serum levels of TC, TG, LDL-C, and HDL-C. (**E**–**G**) Oxidative stress markers, including GSH-Px, SOD, and MDA. Data are presented as mean ± SD (*n* = 6). Different lowercase letters indicate significant differences among groups (*p* < 0.05).

Oxidative stress is a state of imbalance between the body’s oxidation and anti-oxidation systems, often evaluated by measuring antioxidant enzyme activity and lipid peroxidation product levels. The SHR Model group exhibited significant abnormalities in both lipid metabolism and oxidative stress indicators. Compared with the NC group, serum levels of TC, TG, and LDL-C were significantly elevated, while HDL-C levels were markedly decreased in the Model group (Figure 3A–D), suggesting that the hypertensive state was accompanied by significant lipid metabolism disorders. After EULP intervention, all dose groups showed a decreasing trend in TC, TG, and LDL-C levels, while HDL-C levels rebounded, generally restoring them towards normal levels.

To evaluate the effect of EULP on oxidative stress levels in SHRs, the activities of superoxide dismutase (SOD) and glutathione peroxidase (GSH-Px), and the content of malondialdehyde (MDA) were measured in serum. As shown in Figure 3, compared with the NC group, serum SOD and GSH-Px activities were significantly decreased, while MDA content was significantly increased in the Model group SHRs (*p* < 0.05). After 4 weeks of EULP intervention, the activities of SOD and GSH-Px in the PC group and all dose groups (LD, MD, HD) were increased compared to the Model group, while MDA content showed a decreasing trend. Among these, the MD and HD groups showed more significant improvements, with the HD group exhibiting the highest SOD activity and lowest MDA content (*p* < 0.05).

### 3.5. Effects of EULP on Cardiac Inflammatory Protein Expression in SHRs

**Figure 4:**
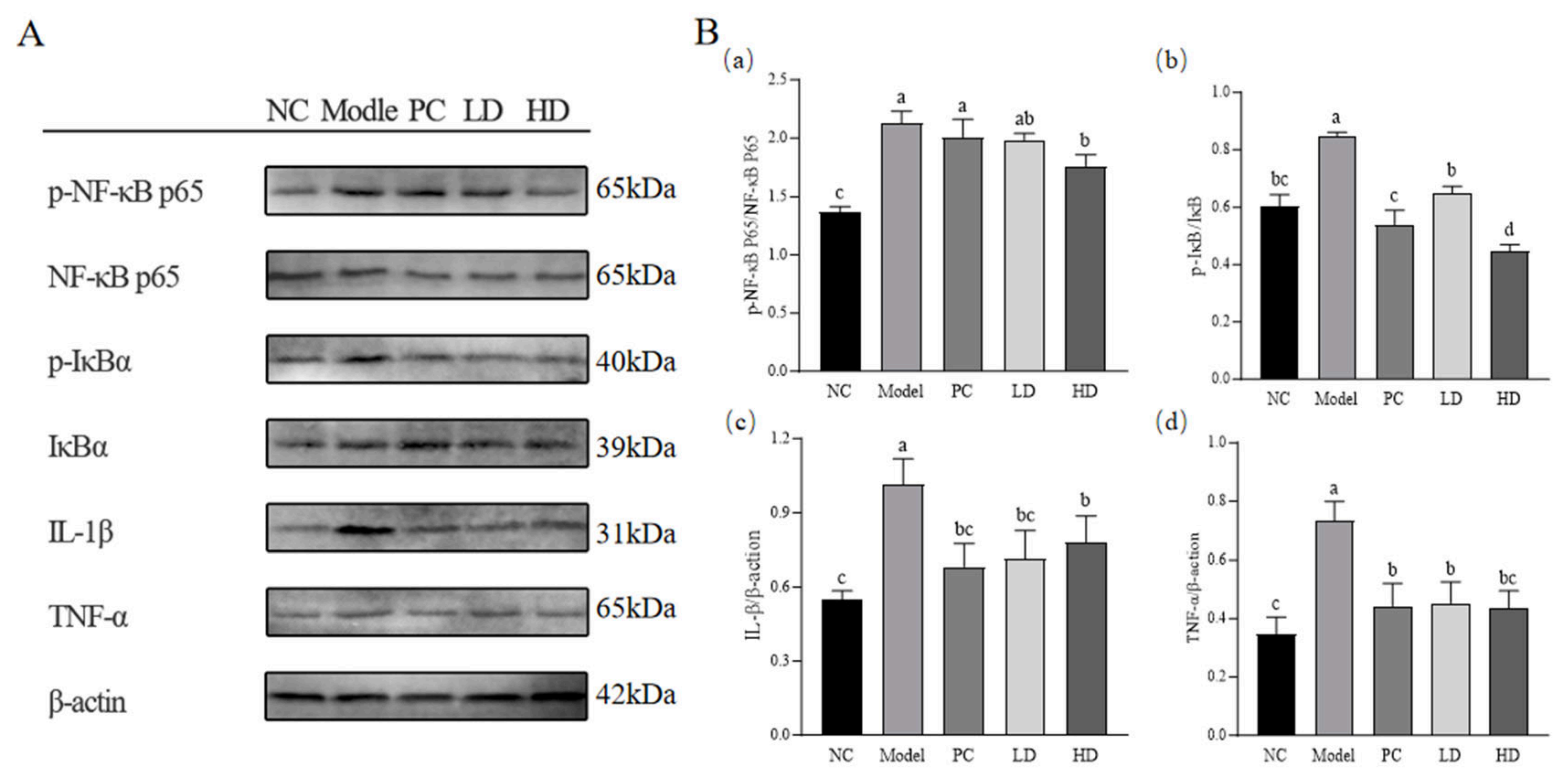
Effects of EULP on NF-κB signaling pathway and pro-inflammatory cytokine expression. (**A**) Western blot analysis of p-NF-κB p65, NF-κB p65, p-IκBα, IκBα, IL-1β, and TNF-α expression. β-actin served as a loading control (Original images are available in the Supplementary Materials). (**B**) Quantitative analysis of (a) p-NF-κB p65/NF-κB p65, (b) p-IκBα/IκBα, (c) IL-1β, and (d) TNF-α levels. Groups: normal control (NC), disease model (Model), positive control (PC), low dose (LD), and high dose (HD) of EULP. Data are presented as mean ± SD (*n* = 3). Different lowercase letters indicate significant differences among groups (*p* < 0.05).

Based on the Western blot bands and their quantitative analysis (Figure 4), distinct differences in the expression of inflammation-related proteins were observed among the groups following EULP intervention. Compared with the NC group, the Model group exhibited significantly increased ratios of p-NF-κB p65/NF-κB p65 and p-IκBα/IκBα, along with elevated expression levels of downstream inflammatory cytokines, including IL-1β and TNF-α. In contrast, the PC group and EULP-treated groups (LD and HD) showed varying degrees of reduction in these indicators compared with the Model group. Notably, the HD group exhibited more pronounced decreases in the ratios of p-NF-κB p65/NF-κB p65 and p-IκBα/IκBα, as well as in IL-1β and TNF-α expression levels, demonstrating a dose-dependent trend. Meanwhile, no significant differences were observed in the total protein levels of NF-κB p65 and IκBα among the groups.

### 3.6. Effects of EULP on Ileal Histopathology and Short-Chain Fatty Acid Levels in SHRs

**Figure 5:**
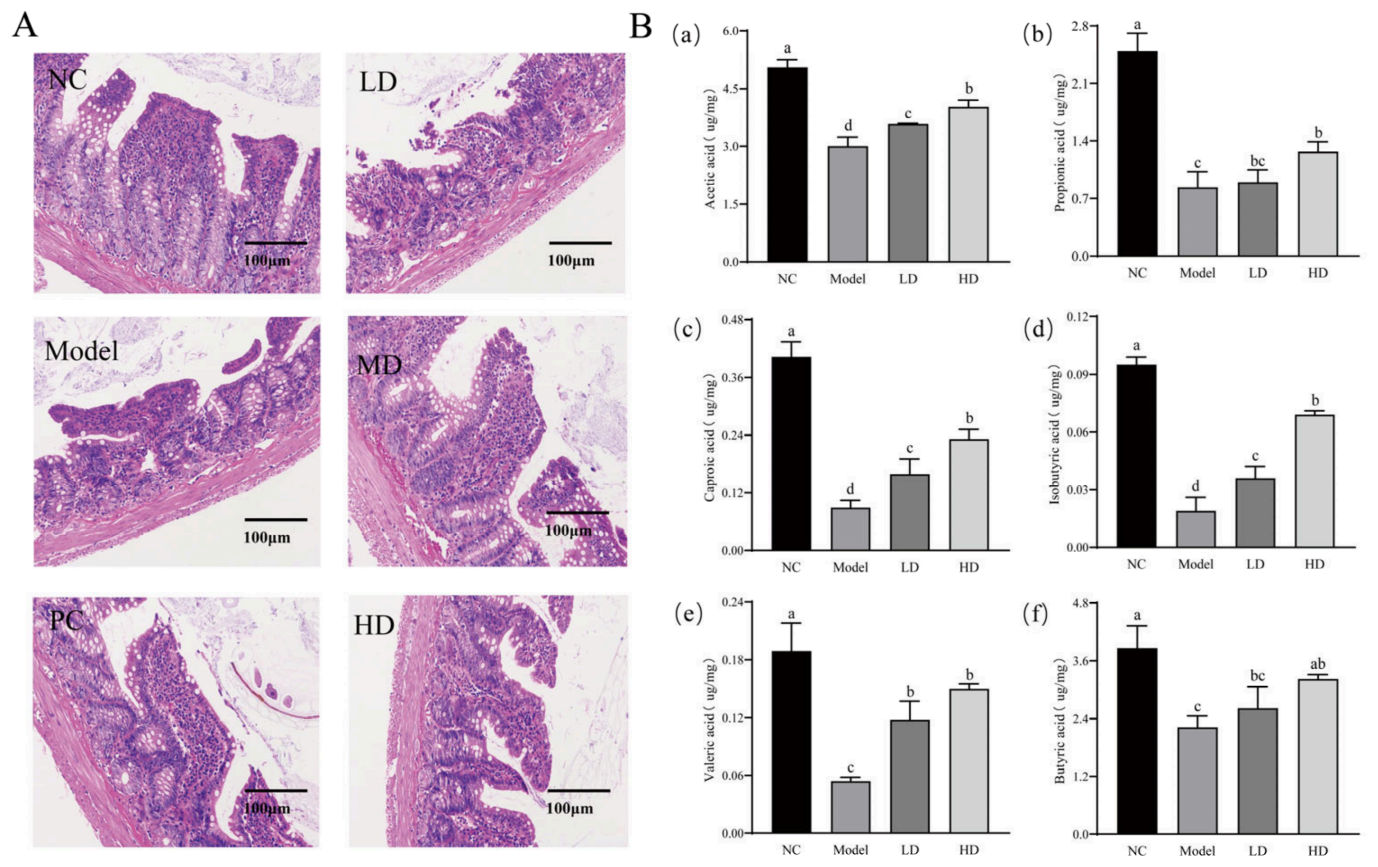
Effects of EULP on intestinal morphology and short-chain fatty acids. (**A**) Representative histological images (H&E staining) of intestinal tissues. Groups: normal control (NC), disease model (Model), positive control (PC), low dose (LD), middle dose (MD), and high dose (HD) of EULP. Scale bar = 100 µm. (**B**) Concentrations of short-chain fatty acids (SCFAs) in intestinal contents, including (a) Acetic acid, (b) Propionic acid, (c) Caproic acid, (d) Isobutyric acid, (e) Valeric acid, and (f) Butyric acid. Groups for SCFA analysis are NC, Model, LD, and HD. Data are presented as mean ± SD (*n* = 6). Different letters above bars indicate statistically significant differences (*p* < 0.05).

Histopathological examination of ileal tissues (Figure 5A) showed that the NC group exhibited intact intestinal architecture with well-organized villi and normal epithelial morphology, consistent with the typical features of healthy intestinal tissue [29]. In contrast, the Model group displayed evident pathological alterations, including disordered villi, epithelial disruption, and inflammatory cell infiltration in the lamina propria. Compared with the Model group, the PC and EULP-treated groups showed varying degrees of improvement in ileal structure, with reduced inflammatory infiltration. Notably, the MD and HD groups exhibited more pronounced protective effects, with tissue morphology approaching that of the NC group, while the LD group showed relatively moderate improvement. These results indicate that EULP alleviates intestinal damage in SHRs.

Based on these observations, fecal short-chain fatty acids (SCFAs), including acetic acid, propionic acid, and butyric acid, were further analyzed (Figure 5B). Compared with the NC group, the Model group showed significantly decreased levels of all three SCFAs (*p* < 0.05). EULP intervention increased SCFA levels in all treated groups, with the most notable elevation observed in the HD group, whereas the LD group showed a weaker effect. These findings suggest that enhanced SCFA production may contribute to the beneficial effects of EULP on intestinal homeostasis and blood pressure regulation.

### 3.7. Effects of EULP on Gut Microbiota in SHRs

#### 3.7.1. Gut Microbial Diversity

**Figure 6:**
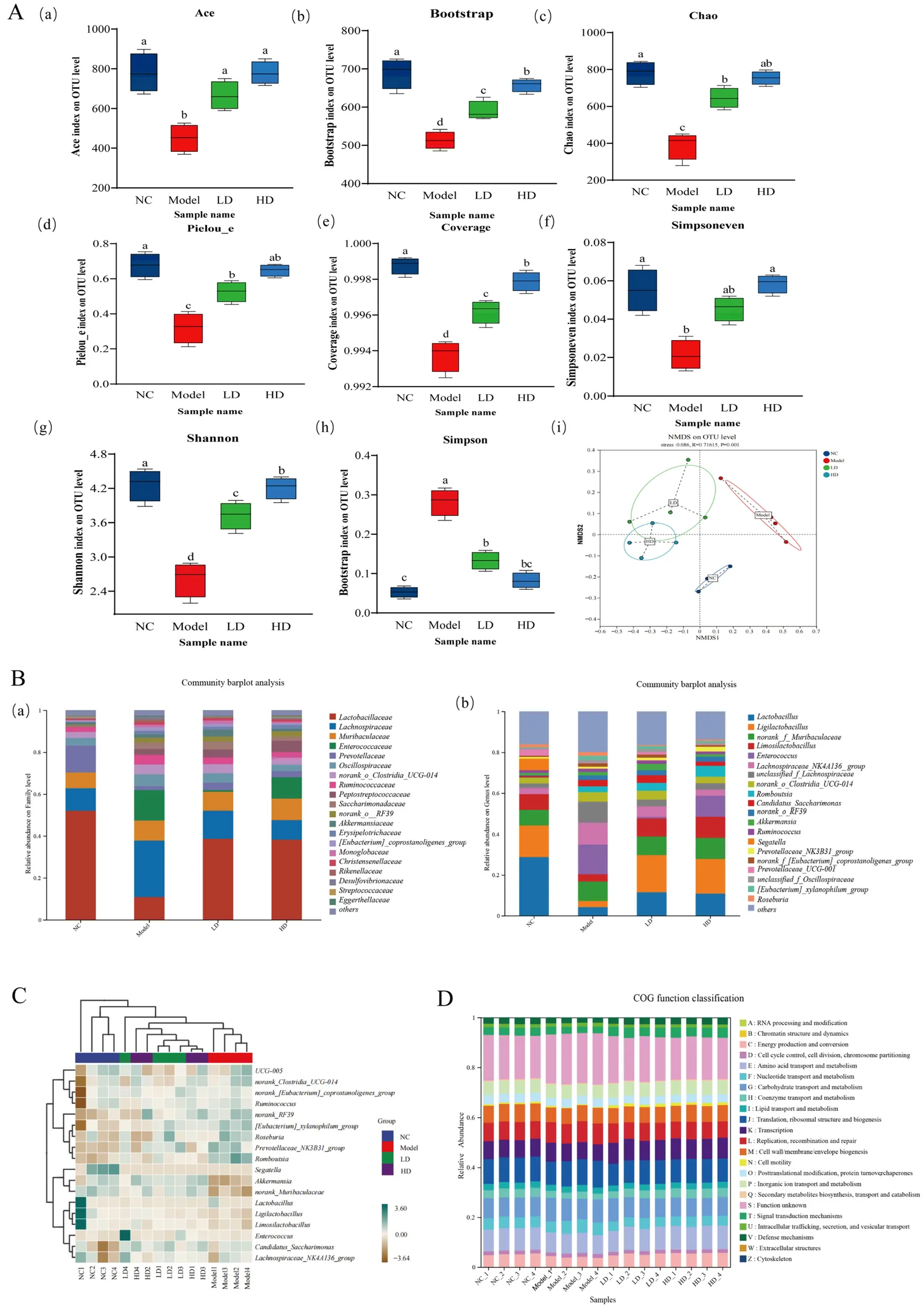
Effects of EULP on gut microbiota composition, diversity, and functional potential. (**A**) Alpha diversity indices: (a) Ace, (b) Bootstrap, (c) Chao, (d) Pielou_e, (e) Coverage, (f) Simpsoneven, (g) Shannon, (h) Simpson, and (i) NMDS plot for beta diversity analysis. (**B**) Relative abundance of gut microbiota at (a) family and (b) genus levels. (**C**) Heatmap illustrating the relative abundance of microbial taxa. (**D**) COG functional classification of the gut microbial community. Experimental groups: normal control (NC), disease model (Model), low dose (LD), and high dose (HD) of EULP. Data are presented as mean ± SD (*n* = 3). Different letters above bars indicate statistically significant differences (*p* < 0.05).

Alpha diversity analysis (Figure 6A) showed that, compared with the NC group, the Model group exhibited significantly reduced richness (Ace, Chao, Bootstrap) and diversity/evenness indices (Shannon, Pielou_e, Simpson evenness), along with an increased Simpson index (*p* < 0.05), indicating decreased microbial diversity. EULP intervention partially restored gut microbial diversity, with significant increases in Ace, Chao, and Shannon indices in the LD and HD groups (*p* < 0.05), particularly in the HD group. Coverage values in all groups exceeded 0.99, confirming sufficient sequencing depth.

Beta diversity analysis based on OTUs revealed clear separation among groups (NMDS: Stress = 0.086, R = 0.71615, *p* = 0.001; Figure 6A(h)). The Model group was distinctly separated from the NC group, whereas EULP-treated groups, especially the HD group, shifted closer to the NC group, indicating partial restoration of microbial structure.

#### 3.7.2. Taxonomic Composition at Family and Genus Levels

At the family level (Figure 6B(a)), the Model group showed decreased abundance of *Lactobacillaceae* and increased *Enterococcaceae* compared with the NC group, indicating microbial dysbiosis. EULP treatment, particularly at high doses, significantly increased *Lactobacillaceae* and suppressed *Enterococcaceae*, restoring the microbial balance. At the genus level (Figure 6B(b)), beneficial bacteria such as *Lactobacillus* and *Ligilactobacillus* were markedly reduced in the Model group, whereas the opportunistic pathogen Enterococcus was enriched. EULP intervention reversed these changes by promoting beneficial bacteria and inhibiting harmful taxa. Additionally, *Akkermansia* and *Muribaculaceae* showed a recovery trend following EULP treatment.

#### 3.7.3. Heatmap Analysis of Genus-Level Abundance

Hierarchical clustering heatmap analysis (Figure 6C) further illustrated microbial differences among groups. The Model group formed a distinct cluster separated from the NC group, whereas the HD group clustered closer to the NC group, indicating restoration of microbial composition. Heatmap patterns showed reduced abundance of beneficial genera (*Lactobacillus*, *Ligilactobacillus*, *Akkermansia*, *Limosilactobacillus*) and enrichment of pathogenic genera (*Enterococcus*, *Romboutsia*, *Candidatus_Saccharimonas*) in the Model group. These alterations were partially reversed by EULP treatment.

#### 3.7.4. Functional Prediction of Gut Microbiota (COG)

Functional prediction using PICRUSt2 (Figure 6D) indicated that gut microbiota were mainly involved in carbohydrate transport and metabolism (G), amino acid transport and metabolism (E), transcription (K), and cell wall/membrane biogenesis (M). Although the overall functional profiles were similar among groups, variations in pathway abundance suggested that EULP modulated microbial metabolic functions by reshaping community composition.

### 3.8. Serum Metabolomics Analysis of SHRs Following EULP Intervention

#### 3.8.1. Global Metabolic Profiling

**Figure 7:**
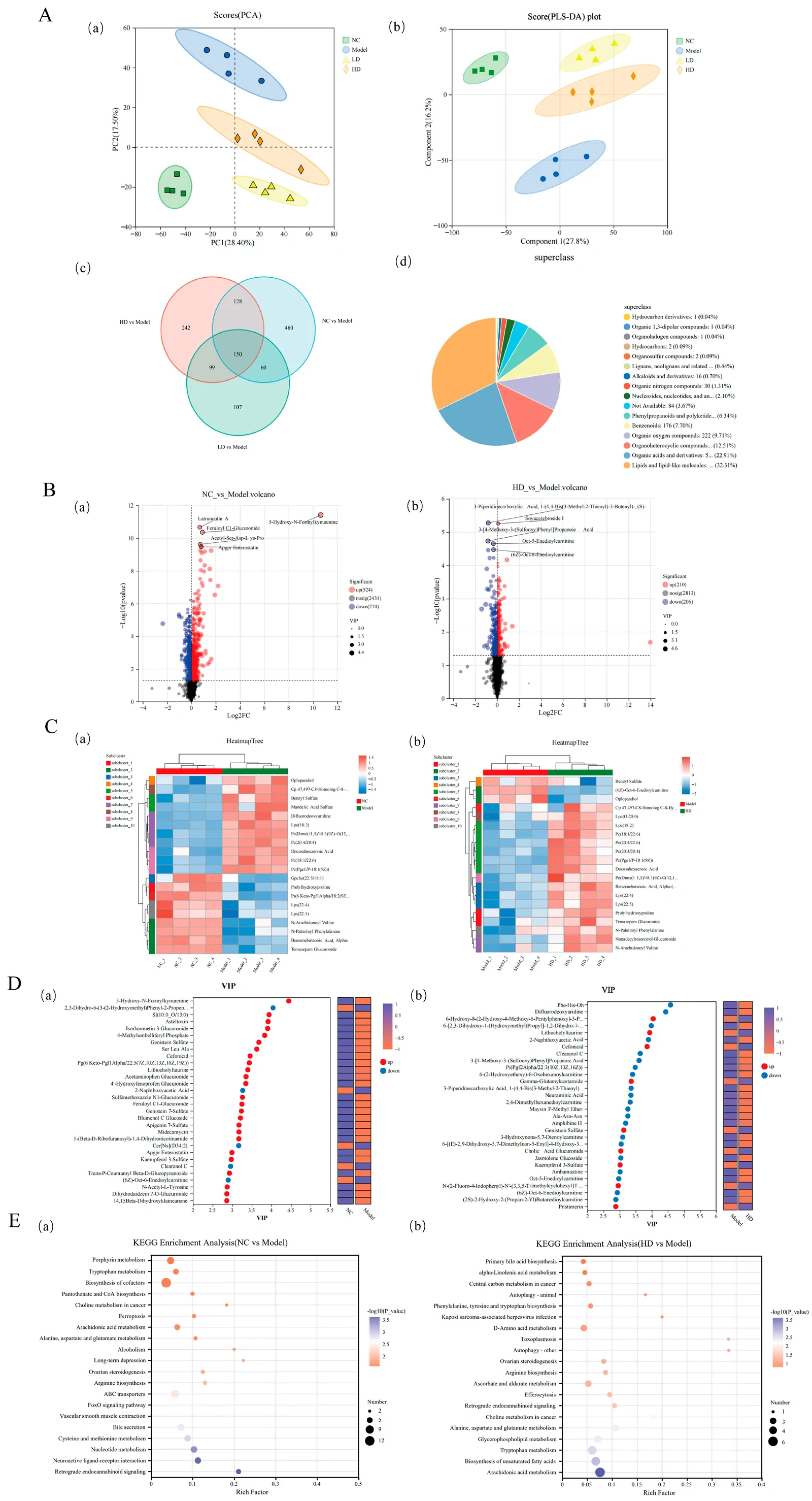
Metabolomics analysis of SHR and effects of EULP. (**A**) Multivariate analysis and metabolite classification: (a) PCA, (b) PLS-DA score plots; (c) Venn diagram of differential metabolites; (d) Metabolite superclass distribution. (**B**) Volcano plots for (a) NC vs. Model and (b) HD vs. Model, showing significantly altered metabolites (red: upregulated, blue: downregulated). (**C**) Heatmaps of relative abundance for differential metabolites in (a) NC vs. Model and (b) HD vs. Model. (**D**) VIP scores and heatmaps of key metabolites for (a) NC vs. Model and (b) HD vs. Model. (**E**) KEGG pathway enrichment analysis for differential metabolites in (a) NC vs. Model and (b) HD vs. Model. Groups: NC (normal control), Model (disease model), LD (low dose), HD (high dose) of EULP. Differential metabolites were identified using VIP > 1.0 and *p* < 0.05. Key differential metabolites were further screened using VIP > 2.0.

Multivariate statistical analyses were performed to evaluate the overall metabolic alterations (Figure 7A). Principal component analysis (PCA) and partial least squares discriminant analysis (PLS-DA) revealed clear separation between the NC and Model groups, indicating significant metabolic disturbances in SHRs. Following EULP intervention, all treatment groups showed a shift toward the NC group, with the HD group exhibiting the most pronounced restoration, suggesting that EULP effectively modulates metabolic homeostasis.

Venn diagram analysis identified 150 common differential metabolites shared among the NC vs. Model, LD vs. Model, and HD vs. Model comparisons (Figure 7A(c)). Metabolite superclass classification (Figure 7A(d)) indicated that these metabolites were mainly classified as lipids and lipid-like molecules (32.31%), organic acids and derivatives (22.91%), and organoheterocyclic compounds (12.51%).

#### 3.8.2. Differential Metabolites: Volcano Plot and Heatmap Analysis

Volcano plot analysis revealed 798 differential metabolites in the NC vs. Model comparison (524 upregulated and 274 downregulated), whereas the number decreased to 416 (210 upregulated and 206 downregulated) in the HD vs. Model comparison (Figure 7B), indicating partial normalization after EULP treatment.

Hierarchical clustering (Figure 7C) further confirmed these findings, with the HD group exhibiting a metabolic profile closer to that of the NC group. Specifically, several phospholipid metabolites, including docosadienoic acid and PE (18:1/22:6), were downregulated in the Model group, whereas metabolites such as benzyl sulfate and mandelic acid sulfate were upregulated. These alterations were partially reversed following EULP intervention.

#### 3.8.3. Key Metabolites and Pathway Enrichment Analysis

Key differential metabolites were further screened based on variable importance in projection (VIP > 2.0) from the PLS-DA model(Figure 7D). Among them, 5-hydroxy-N-formylkynurenine exhibited a high VIP value (VIP > 5.5) in the NC vs. Model comparison, indicating its strong discriminatory power. Additionally, lithocholytaurine showed consistently high VIP values in both NC vs. Model and HD vs. Model comparisons.

KEGG pathway enrichment analysis (Figure 7E) revealed that differential metabolites in the NC vs. Model comparison were mainly enriched in porphyrin metabolism, tryptophan metabolism, and arachidonic acid metabolism. In contrast, those in the HD vs. Model comparison were primarily enriched in primary bile acid biosynthesis, α-linolenic acid metabolism, and tryptophan metabolism.

Overall, EULP-regulated metabolic pathways mainly involved arachidonic acid metabolism, biosynthesis of unsaturated fatty acids, and tryptophan metabolism, along with key metabolites such as spermine, adenosine, and indole-3-acetic acid (Table 1). These results suggest that EULP exerts antihypertensive effects by modulating lipid and amino acid metabolism.

### 3.9. Spearman Correlation Analysis and Mechanistic Insights

**Figure 8:**
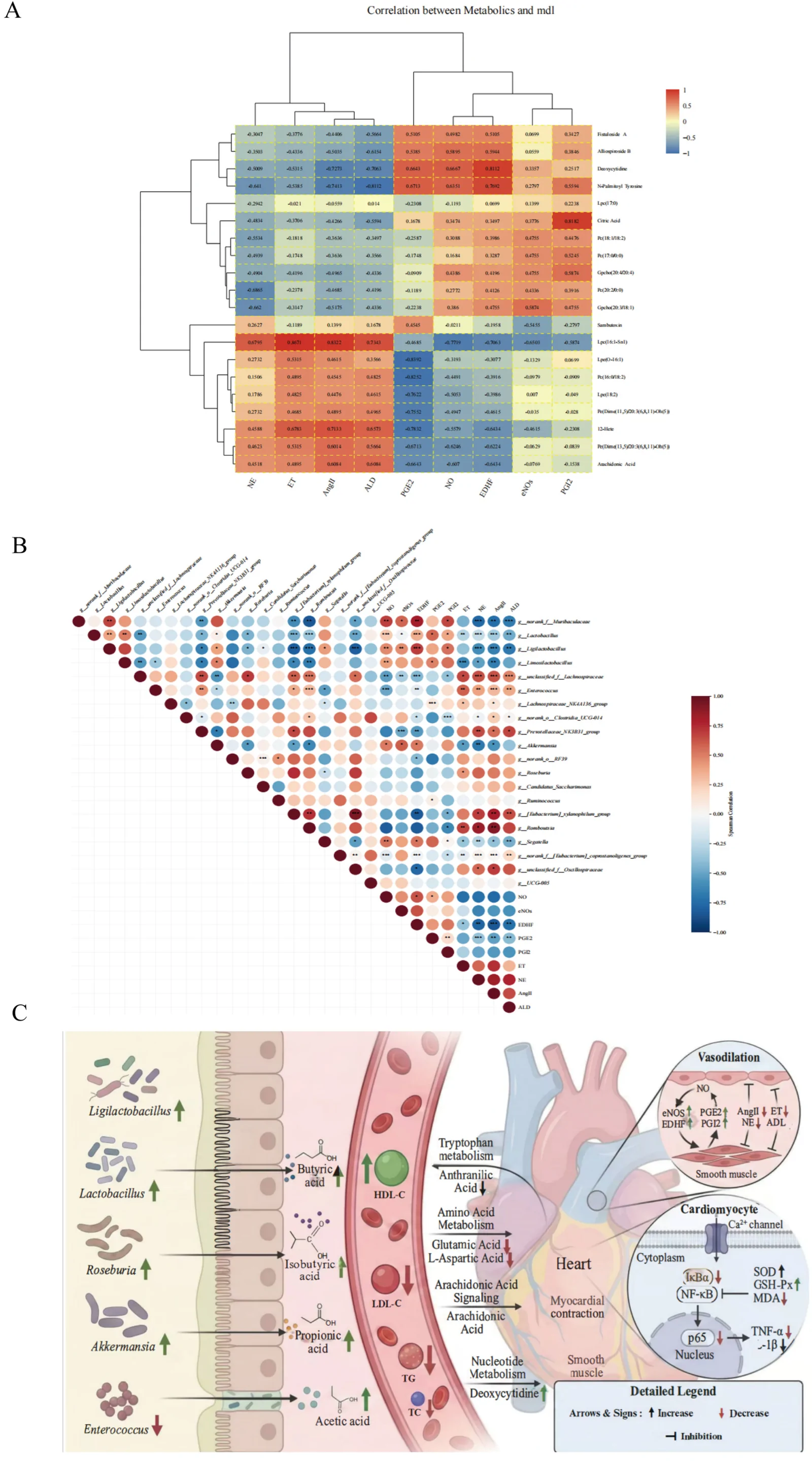
Schematic representation of the gut microbiota–metabolite–cardiovascular axis and correlation analyses. (**A**) Correlation heatmap between identified metabolites and md. (**B**) Correlation heatmap depicting relationships among different microbial groups. * *p* < 0.05, ** *p* < 0.01, and *** *p* < 0.001. (**C**) Diagram illustrating the gut–heart axis, showing the influence of specific gut microbiota (e.g., *Ligilactobacillus*, *Lactobacillus*, *Roseburia*, *Akkermansia*, *Enterococcus*) on short-chain fatty acid (SCFA) production (Butyric, Isobutyric, Propionic, Acetic acids). These metabolites affect cardiac function via modulation of vasodilation (e.g., NO, eNOS, Ang II), amino acid metabolism, arachidonic acid signaling, and cardiomyocyte inflammatory/oxidative stress responses (e.g., TNF-α, IL-1β, MDA).

To further elucidate the underlying mechanisms, Spearman correlation analysis was performed to assess the relationships among gut microbiota, differential metabolites, and vascular regulatory factors (Figure 8). Correlation heatmap analysis (Figure 8A) showed that lipid metabolites, including arachidonic acid and 12-HETE, were positively correlated with vasoconstrictive factors (Ang II, NE, ET, ALD, and PGE2; r > 0.6), but negatively correlated with vasodilatory indicators (NO, eNOS, EDHF, and PGI_2_).

Further analysis (Figure 8B) revealed that beneficial genera such as Akkermansia, Roseburia, and Lactobacillus were positively associated with vasodilatory factors (e.g., NO and eNOS) and negatively associated with vasoconstrictive factors (e.g., Ang II and ALD). These findings highlight a coordinated regulatory network linking gut microbiota, metabolites, and vascular function, supporting the role of the gut microbiota–metabolite axis in EULP-mediated antihypertensive effects.

### 3.10. Identification of Polyphenolic Compounds in EULP

To characterize the chemical composition of EULP, GC-MS analysis was performed following solid-phase extraction. Based on retention time, mass spectral fragmentation patterns, and comparison with the NIST database and literature retention indices, a total of 18 polyphenolic compounds were preliminarily identified (Table 2). Among them, compounds such as homovanillyl alcohol, 4-hydroxybenzoic acid, syringic acid, and 3-(4-hydroxy-3-methoxyphenyl) propionic acid are typical products of the phenylpropanoid metabolic pathway, indicating the chemical diversity and bioactive potential of EULP.

## 4. Discussion

Hypertension is a multifactorial disorder involving vascular endothelial dysfunction, chronic inflammation, oxidative stress, gut microbial dysbiosis, and metabolic disturbances [30,31]. Recent studies have highlighted the importance of the gut microbiota–metabolite–host axis in blood pressure regulation, in which microbial metabolites participate in the modulation of vascular homeostasis, immune responses, and host metabolism [32]. As a traditional edible and medicinal resource, *Eucommia ulmoides* leaves contain abundant polyphenolic compounds with potential cardiovascular benefits. In the present study, EULP intervention markedly reduced blood pressure and attenuated cardiac injury in SHRs, accompanied by improvements in gut microbial composition and host metabolic profiles. These findings suggest that the antihypertensive effects of EULP may involve coordinated interactions between gut microbiota and host metabolic regulation rather than a single-target mechanism.

The regulation of vascular tone depends on the balance between vasodilatory and vasoconstrictive factors, and disruption of this balance is a central mechanism in hypertension [33,34]. Nitric oxide (NO), primarily produced by endothelial nitric oxide synthase (eNOS), plays a key role in maintaining vascular homeostasis together with EDHF and PGE2 [35,36,37,38]. In contrast, endothelin (ET) induces sustained vasoconstriction via ETA receptor-mediated Ca^2+^ influx [39]. Angiotensin II (Ang II) further elevates blood pressure by stimulating aldosterone (ALD) secretion and increasing blood volume, acting synergistically with norepinephrine (NE) [40]. In this study, EULP significantly increased vasodilatory factors while suppressing vasoconstrictive mediators, thereby improving endothelial function and vascular homeostasis. These findings are consistent with previous reports highlighting the pivotal role of the NO/eNOS pathway in vascular regulation [41].

Beyond regulating vascular tone, EULP attenuated cardiovascular injury, potentially by interrupting the vicious cycle between oxidative stress and inflammation, a key driver of hypertension progression [42,43]. Oxidative stress, inflammation, and dyslipidemia collectively contribute to endothelial dysfunction and vascular damage, while sustained hypertension further accelerates these pathological processes [44]. In the present study, SHRs exhibited enhanced lipid peroxidation and impaired antioxidant capacity [45,46], accompanied by elevated expression of pro-inflammatory cytokines such as TNF-α and IL-1β in cardiac tissue. EULP intervention significantly restored antioxidant status and reduced inflammatory protein expression. Given the known regulatory effects of polyphenols on NF-κB signaling [47,48], these results suggest that EULP exerts cardioprotective effects potentially by enhancing antioxidant defenses and inhibiting pro-inflammatory pathways.

Regulation of blood pressure and inflammation is also closely linked to gut microbiota and their metabolites [49,50,51]. In this study, EULP significantly restored both α- and β-diversity of gut microbiota in SHRs and selectively enriched beneficial genera such as *Akkermansia*, *Roseburia*, and *Lactobacillus* [52,53,54]. These microbial changes were accompanied by increased SCFA production, which we hypothesize may contribute to intestinal homeostasis and vascular regulation. SCFAs serve as key energy sources for intestinal epithelial cells and possess anti-inflammatory properties, contributing to the maintenance of gut barrier integrity and systemic metabolic balance [55,56,57]. Thus, EULP may regulate systemic blood pressure in part by promoting SCFA-producing bacteria and improving gut-derived metabolic signaling.

In this study, serum metabolomics showed obvious alterations in lipid and amino acid metabolism in SHRs, whereas EULP intervention partially restored these metabolic disturbances. The serum metabolic profile of SHRs was markedly altered compared with the NC group, while EULP intervention partially normalized these changes, particularly in lipid and amino acid metabolism. Notably, arachidonic acid metabolites (e.g., HETE) are known to promote vasoconstriction and amplify inflammatory responses [58], and were significantly downregulated following EULP treatment. In addition, EULP modulated bile acid metabolism, including lithocholytaurine, which can regulate inflammation and energy homeostasis via FXR and TGR5 signaling pathways [59,60]. These findings further support that EULP alleviates hypertension potentially through regulation of metabolic pathways related to inflammation and vascular function.

To further integrate these findings, Spearman correlation analysis revealed strong associations among gut microbiota, metabolites, and vascular regulatory factors, suggesting potential interactions among gut microbiota, metabolites, and vascular factors [61]. Changes in microbial composition may influence host metabolism, which in turn regulates vasoactive factors and blood pressure.

Based on these multi-omics results, we propose a potential mechanistic model underlying the antihypertensive effects of EULP (Figure 8C). In this potential model, EULP was associated with remodeling of gut microbiota, including enrichment of SCFA-producing bacteria such as *Lactobacillus* and *Roseburia*, together with alterations in key metabolic pathways, including arachidonic acid, tryptophan, and nucleotide metabolism. These microbiota-associated metabolic alterations may contribute to improved vascular homeostasis and reduced inflammatory responses in SHRs.

Several limitations should be acknowledged. First, the lack of fecal microbiota transplantation or antibiotic intervention experiments limits the establishment of a causal relationship between gut microbiota and blood pressure reduction. Second, some analyses used *n* = 6, and the PC and MD groups were omitted from selected analyses, which may have reduced statistical power. Third, the polyphenolic compounds were tentatively identified by GC-MS spectral library matching without authentic standards. Future studies should further validate these findings using targeted and causality-based approaches.

Collectively, the current findings suggest that EULP may exert antihypertensive effects associated with modulation of the gut microbiota–metabolite axis, highlighting its potential as a multi-target functional food strategy for the prevention and management of hypertension and cardiovascular remodeling. However, the causal relationships between gut microbiota and host metabolic alterations require further investigation. In addition, the active polyphenolic constituents responsible for these effects remain to be further identified.

## 5. Conclusions

In summary, EULP exhibited significant antihypertensive effects in SHRs, and these beneficial effects were associated with improvements in vascular dysfunction, suppressing oxidative stress and inflammation, and restoring gut microbial homeostasis. Mechanistically, EULP enhanced the abundance of beneficial bacteria and promoted SCFA production, accompanied by the modulation of key metabolic pathways associated with arachidonic acid metabolism, tryptophan metabolism, and bile acid biosynthesis. These microbiota-derived metabolic alterations were closely associated with improved vascular regulatory balance and reduced inflammatory signaling. Collectively, these findings suggest that the antihypertensive effects of EULP are potentially linked to modulation of the gut microbiota and metabolic profiles. However, these findings indicate associations rather than definitive causality, and further studies are needed to validate the underlying mechanisms and identify the key bioactive compounds.

## Figures and Tables

**Table 1:** Key metabolic pathways and differential metabolites associated with EULP.

Control Group	NC vs. Model	HD vs. Model
The top 5 metabolic pathways	Retrograde endocannabinoid signaling	Arachidonic acid metabolism
	Neuroactive ligand-receptor interaction	Biosynthesis of unsaturated fatty acids
	Nucleotide metabolism	Tryptophan metabolism
	Cysteine and methionine metabolism	Glycerophospholipid metabolism
	Bile secretion	Alanine, aspartate, and glutamate metabolism
The top 20 differential metabolites	Spermine	N-Acetylserotonin
	Deoxycytidine	N-Acetyl-L-Aspartic Acid
	Adenosine	15-Deoxy-Delta-12,14-Prostaglandin J2
	Ouabain	5-Oxoete
	Gpcho (20:1/20:4)	12-Oxo-Ete
	Gpcho (22:5/16:1)	Gpcho (20:1/20:4)
	Microcystin-Lr	Pe (14:0/22:4)
	Glutamic Acid	Gpcho (22:5/16:1)
	Adp	11,12-Epoxyeicosatrienoic Acid
	Morph	Indole-3-Acetic Acid
	Cytidine	Anthranilic Acid
	O-Acetylserine	Argininosuccinic Acid
	S-Adenosylhomocysteine	L-Serine
	Thymidine	Citric Acid
	5′-Methylthioadenosine	Lpc (20:2)
	Endomorphin-1	Lpc (17:0)
	Pe(36:4)	11Z-Eicosenoic Acid
	Pe (P-16:0/22:6)	Docosatetraenoic Acid
	5-Methylthioribose	Lpc (20:0)
	Pc (15:0/22:6)	Pe (36:4)

Differential metabolites were screened based on VIP values (VIP > 2.0) and statistical significance (*p* < 0.05). Pathway enrichment analysis was conducted using the KEGG database. NC, normal control group; Model, hypertensive model group; HD, high-dose EULP group.

**Table 2:** GC–MS identification of polyphenolic compounds in EULP.

NO	Compound Name	Molecular Formula	CAS	Relative Content (%)	Retention Time (min)
1	1,3,5-Benzetriol, 3TMS derivative	C_6_H_6_O_3_	10586-12-6	0.30	2.53
2	1,3-Benzenedicarboxylic acid, 5-(1,1-dimethylethyl)-	C_12_H_14_O_4_	2359-9-3	4.22	6.99
3	Homovanillyl alcohol, 2TMS derivative	C_9_H_12_O_3_	56728-6-4	4.92	12.09
4	3-(4-Hydroxy-3-methoxyphenyl)propionic acid	C_10_H_12_O_4_	56051-49-1	0.44	15.13
5	2,4-Dihydroxybenzaldehyde	C_7_H_6_O_3_	33617-38-8	0.11	15.22
6	4-Hydroxybenzoic acid	C_7_H_6_O_3_	2078-13-9	0.17	23.53
7	Syringic acid	C_9_H_10_O_5_	10517-29-0	0.54	26.06
8	3-Hydroxymandelic acid	C_8_H_8_O_4_	68595-69-7	0.28	26.35
9	Thymol	C_10_H_14_O	55012-80-1	0.20	30.50
10	2,3-Dihydroxybenzoic acid	C_7_H_6_O_4_	3618-19-7	0.10	35.53
11	Phenol, 3-methyl-5-(1-methylethyl)-, methylcarbamate	C_12_H_16_NO_2_	2631-37-0	2.25	40.53
12	Phenol, 2,6-dimethoxy-	C_8_H_10_O_3_	2005-03-27	3.17	42.58
13	2,4-Di-tert-butylphenol	C_14_H_22_O	96-76-4	8.11	43.47
14	Phenol, 2-methoxy-4-(1-propenyl)-, (Z)-	C_10_H_12_O_2_	5912-86-7	1.50	45.20
15	(E)-1-(2-Hydroxy-4,6-dimethoxyphenyl)-3-phenylprop-2-en-1-one	C_17_H_16_O_4_	1775-97-9	0.12	45.36
16	2,4,7,9-Tetramethyl-5-decyn-4,7-diol	C_14_H_26_O_2_	126-86-3	0.11	47.32
17	2H-1-Benzopyran-2-one, 4-methyl-	C_10_H_8_O_2_	607-71-6	0.12	47.47
18	1,2-Cyclohexanediol, 1-phenyl-, trans-	C_8_H_14_O_3_	27167-34-6	0.67	52.74

Compound assignments were based on comparison of the acquired EI mass spectra with reference spectra in the NIST mass spectral library. The detected compounds were therefore considered tentatively identified based on spectral-library matching. Relative content (%) was calculated by peak area normalization.

## Data Availability

The original contributions presented in this study are included in the article/Supplementary Materials. Further inquiries can be directed to the corresponding author.

## Supplementary Materials

The following supporting information can be downloaded at: https://www.mdpi.com/article/10.3390/foods15173032/s1, Figure S1: Full, uncropped western blot images corresponding to Figure 4A in the main text; Figure S2: Total ion chromatogram (TIC) of polyphenolic compounds in Eucommia ulmoides leaf extract analyzed by GC-MS.
