# Supplementary material for: Eucommia ulmoides Leaf Polyphenols Alleviate Hypertension and Modulate Gut Microbiota and Metabolic Profiles in Spontaneously Hypertensive Rats
Source: Foods. 2026 Aug 27;15(17):3032. doi: 10.3390/foods15173032 (PMC13565005; doi:10.3390/foods15173032)
Supplement: Supplementary file 1 [file foods-15-03032-s001.zip › foods-4469530-supplementary.pdf]

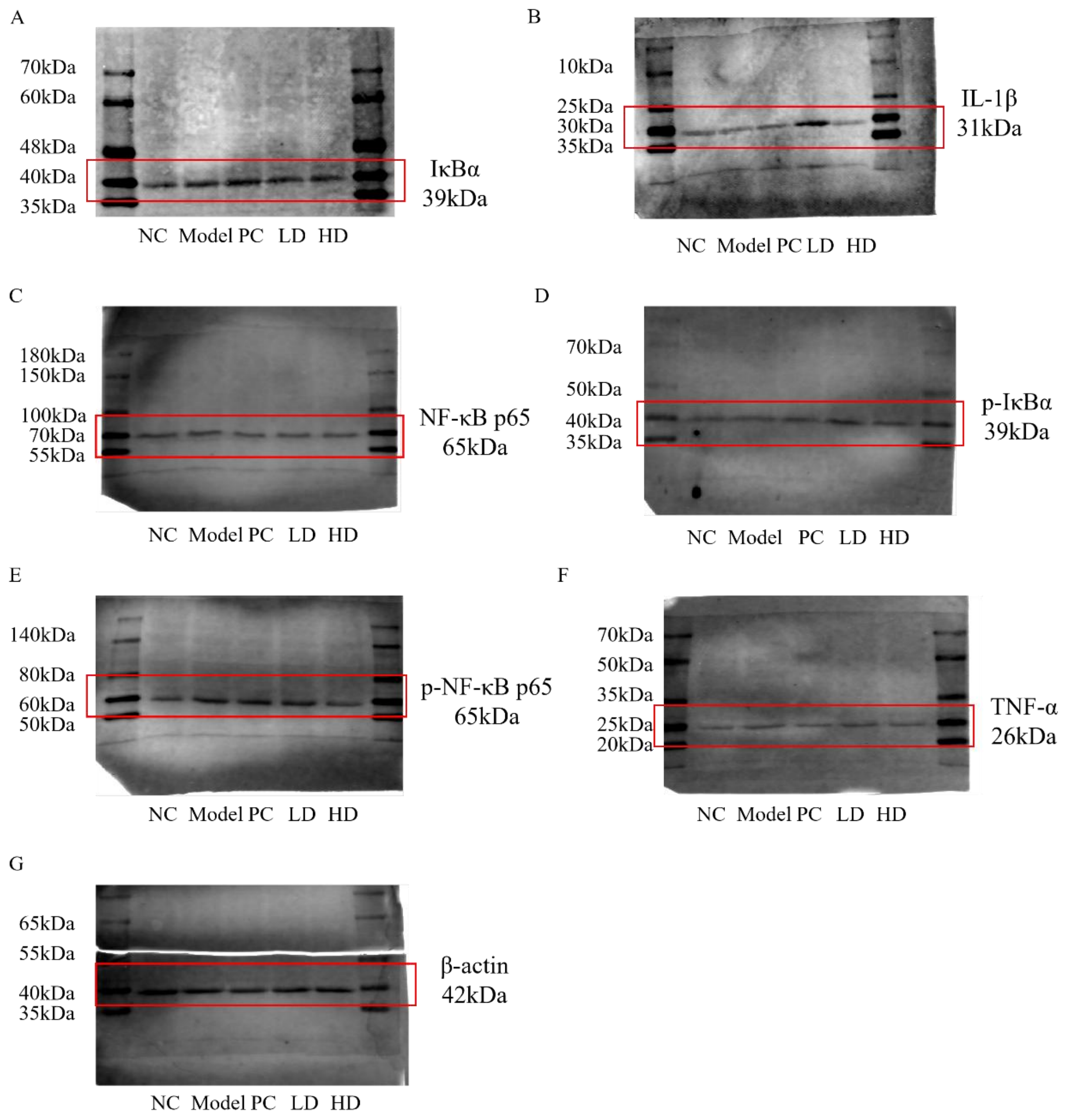

Figure S1. Full, uncropped western blot images corresponding to Figure 4A in the main text.

The membrane was probed with the following antibodies: (A) IκBα, (B) IL-1β, (C) NF-κB p65, (D) p-IκBα, (E) p-NF-κB p65, (F) TNF-α, and (G) β-actin (loading control). The prestained protein ladder (molecular weight marker) is shown on the left lane. Sample loading order from left to right after the marker: NC group, Model group, PC group, LD group, HD group.

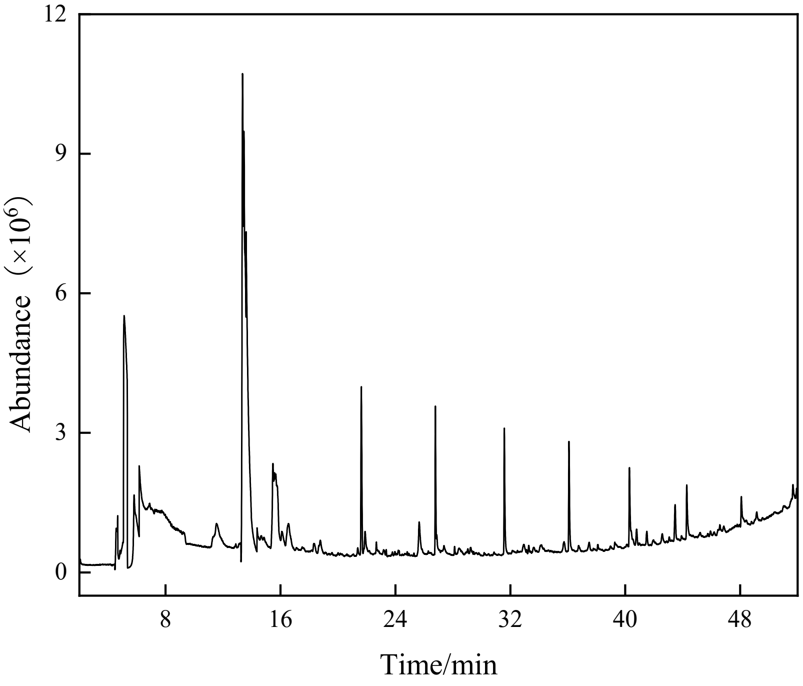

Figure S2. Total ion chromatogram (TIC) of polyphenolic compounds in Eucommia ulmoides leaf extract analyzed by GC-MS.

The chromatogram shows the separation profile of major compounds identified in the extract. Peaks were assigned based on retention time and mass spectral matching with the NIST library.
